# Supplementary material for: Rab Geranylgeranyltransferase Subunit Beta as a Potential Indicator to Assess the Progression of Amyotrophic Lateral Sclerosis
Source: Brain Sci. 2023 Oct 30;13(11):1531. doi: 10.3390/brainsci13111531 (PMC10670085; doi:10.3390/brainsci13111531)

Rab Geranylgeranyltransferase Subunit Beta as a Potential Indicator to Assess the Progression of Amyotrophic Lateral Sclerosis

Figure S1. Correlation between Amyotrophic Lateral Sclerosis Functional Rating Scale–Revised (ALSFRS-R) score and BMI, serum CRP levels, disease duration, and serum IL-6 levels at diagnosis.

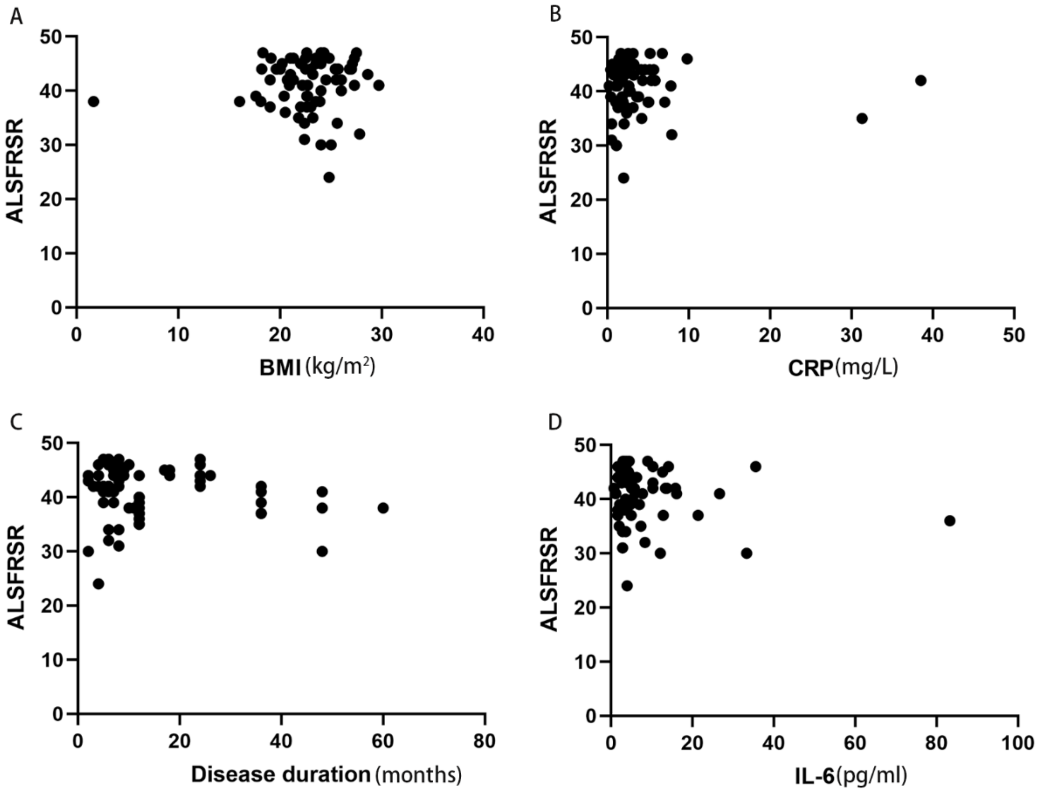

Figure S2. Correlation between progression rate (Delta-FS) of Amyotrophic Lateral Sclerosis and BMI, serum CRP levels, serum IL-6 levels, and age at diagnosis.

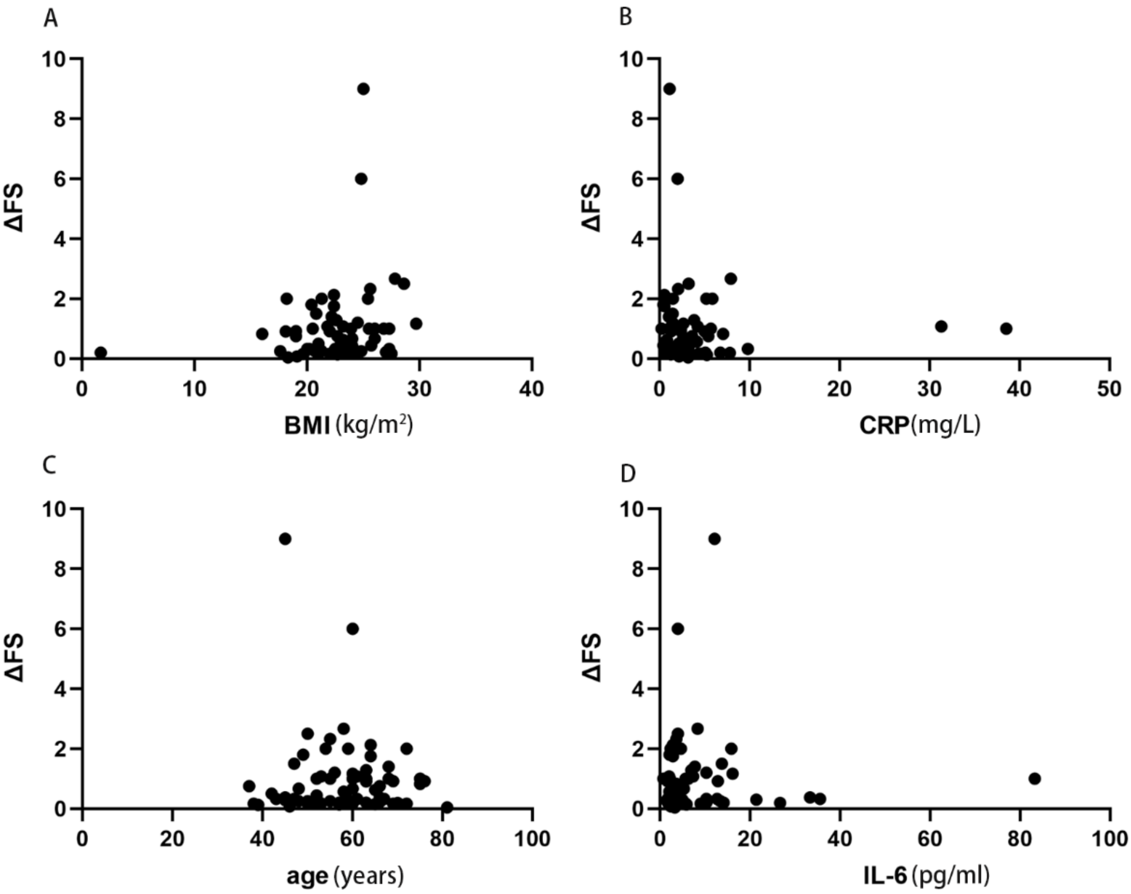

Figure S3. Correlation between Amyotrophic Lateral Sclerosis Functional Rating Scale–Revised (ALSFRS-R) score and BMI, serum CRP levels, disease duration, serum IL-6 levels, and age at diagnosis.

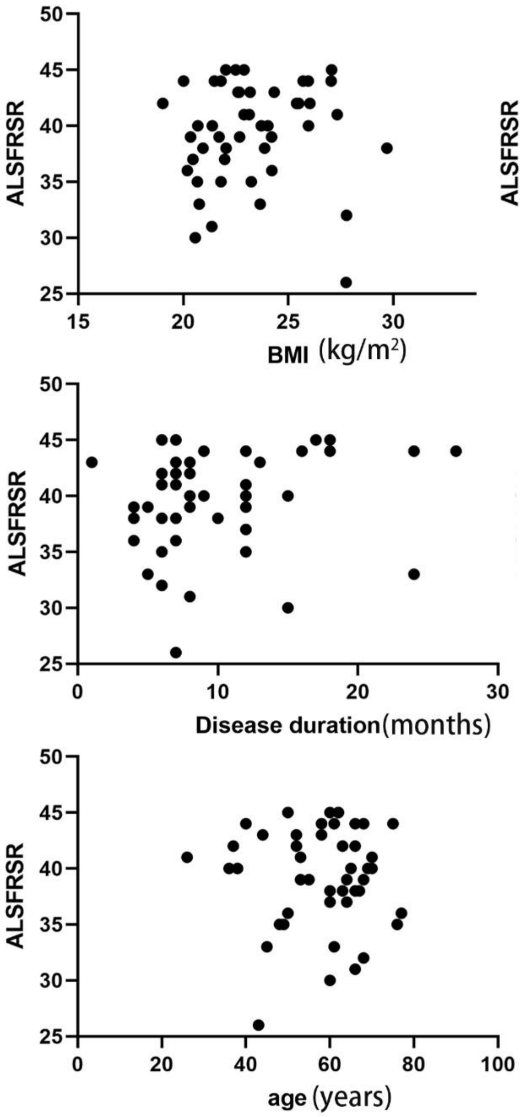

Figure S4. Correlation between progression rate (Delta-FS) of Amyotrophic Lateral Sclerosis and BMI, serum CRP levels, serum IL-6 levels, and age at diagnosis.

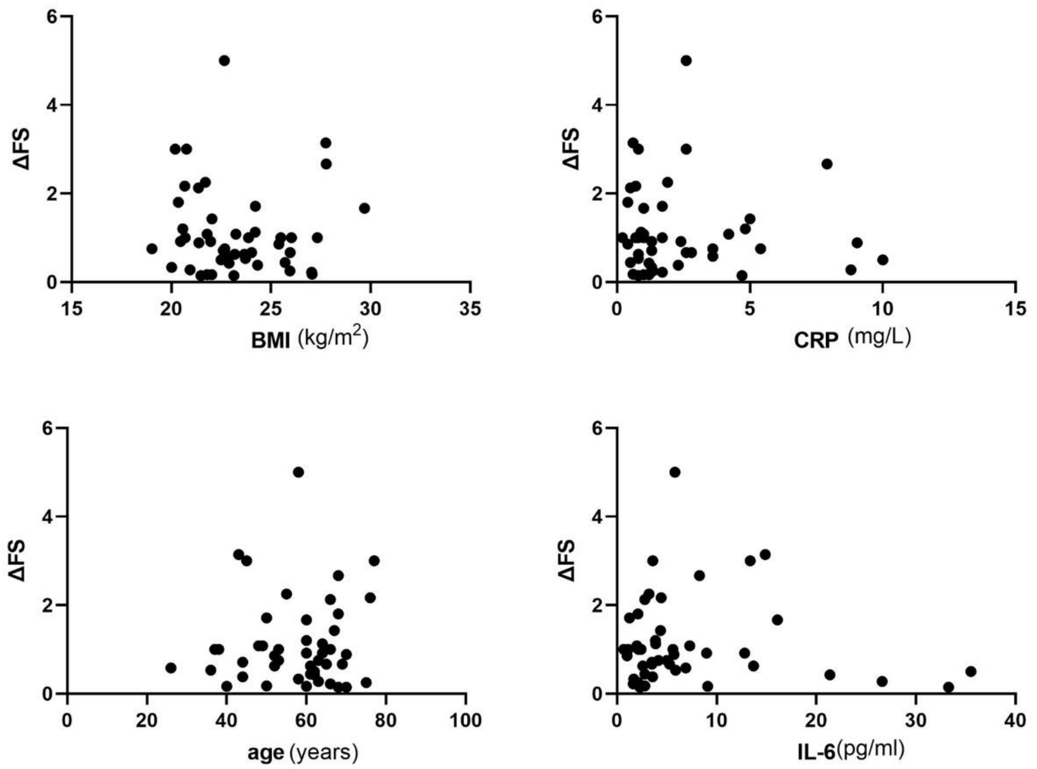

Supplement: Supplementary file 1 [file brainsci-13-01531-s001.zip › brainsci-2613131-supplementary.pdf]
